# High Doses of Caffeine during the Peripubertal Period in the Rat Impair the Growth and Function of the Testis

**DOI:** 10.1155/2015/368475

**Published:** 2015-04-23

**Authors:** Minji Park, Yuri Choi, Hyeonhae Choi, Ju-Yearn Yim, Jaesook Roh

**Affiliations:** Laboratory of Reproductive Endocrinology, Department of Anatomy & Cell Biology, College of Medicine, Hanyang University, Seoul 133-791, Republic of Korea

## Abstract

Prenatal caffeine exposure adversely affects the development of the reproductive organs of male rat offspring. Thus, it is conceivable that peripubertal caffeine exposure would also influence physiologic gonadal changes and function during this critical period for sexual maturation. This study investigated the impact of high doses of caffeine on the testes of prepubertal male rats. A total of 45 immature male rats were divided randomly into three groups: a control group and 2 groups fed 120 and 180 mg/kg/day of caffeine, respectively, via the stomach for 4 weeks. Caffeine caused a significant decrease in body weight gain, accompanied by proportional decreases in lean body mass and body fat. The caffeine-fed animals had smaller and lighter testes than those of the control that were accompanied by negative influences on the histologic parameters of the testes. In addition, stimulated-testosterone ex vivo production was reduced in Leydig cells retrieved from the caffeine-fed animals. Our results demonstrate that peripubertal caffeine consumption can interfere with the maturation and function of the testis, possibly by interrupting endogenous testosterone secretion and reducing the sensitivity of Leydig cells to gonadotrophic stimulation. In addition, we confirmed that pubertal administration of caffeine reduced testis growth and altered testis histomorphology.

## 1. Introduction

High caffeine containing energy drinks are widely consumed by adolescents [1]. Due to the risk-taking behavior of adolescents and their lack of education on the negative effects of caffeine, cases of toxicity due to the consumption of energy drinks are likely to continue to increase [1–3]. In particular, there is growing concern about a decrease in male reproductive health, because a number of laboratory animal studies and human cases have pointed to toxic effects of caffeine on the male reproductive system [3–7]. For instance, caffeine induced testicular atrophy in adult animals, resulting in decreased spermatogenesis [8]. In addition, gestational and lactational exposure of rats to caffeine had adverse effects on the size and structure of the testis, serum testosterone levels, and the fertility of male offspring [3]. Since the adolescent gonad is not fully mature either anatomically or histochemically [9], it would not be surprising if chronic exposure to caffeine during this vulnerable period were to interfere with normal gonad maturation. Though detrimental effects of caffeine on the development of the reproductive system during prenatal period have been widely demonstrated, data on the effect on the reproductive system during the prepubertal period are relatively sparse and conflicting.

The purpose of this study was to investigate the effects of caffeine on the parameters of growth and maturation of the testis in prepubertal male rats throughout most of their rapid growth period. We assessed changes in the gross and microscopic structure of the testis along with changes in body composition caused by caffeine exposure. In addition, effects of caffeine on serum testosterone, known to be important for pubertal sexual maturation, were evaluated, and ex vivo testosterone production was measured in Leydig cells retrieved from testes after caffeine exposure to assess their secretory activity.

## 2. Materials and Methods

### 2.1. Animal

Sprague-Dawley (SD) male rats (*n* = 45) aged two weeks were obtained from Samtako Biokorea (Kyunggi, South Korea). The rats were allowed to acclimate until 21 days of age before being used in experiments. Each animal was housed in a separate plastic cage under controlled conditions (22–24°C, humidity 40–50%, and 12-h light-dark cycle) with free access to food and water. Animal care was consistent with institutional guidelines, and the Hanyang University ACUC committee approved all procedures involving animals (HY-IACUC-2013-0110A).

### 2.2. Experimental Design

The experiment was started when the rats were 22 days of age, because postnatal days 22–25 are considered the beginning of puberty in the rat [10]. Rats were assigned to groups by the stratified randomization method based upon body weight on the day before the start of treatment, in order to eliminate variation in mean body weight between the groups. Rats weighed approximately 50 ± 2 g on the first day of the experiment. Caffeine (C0750, Sigma-Aldrich, St. Louis, MO, USA) was dissolved in distilled water (10 mL/kg) at concentrations calculated to deliver 120- and 180-mg/kg body weight/day (designated as CF1 and CF2, *N* = 15/group). Animals were fed caffeine dissolved in water by gavage to ensure complete consumption of the established dose, once daily in the morning (9 to 11 am). Fifteen animals in the control group (CTRL) received the same volume of distilled water used as vehicle. A treatment duration of 4 weeks was selected to cover the time of rapid growth of the reproductive system of rats (from 22 days to 55 days) [11]. Animals were examined for clinical signs and weighed on a daily basis, and food intake was also monitored. Body weight was measured to the nearest 0.1 g with an electronic scale (Dretec Corp., Seoul, Korea) and recorded on the first day before feeding caffeine (initial) until the day they were killed (final). The caffeine and control experiments were run in parallel. All the animals were killed 24 h after the last treatment following protocols and ethical procedures. They were anesthetized by isoflurane inhalation (Forane solution, Choongwae Pharma Corp., Seoul, Korea) and killed by cervical dislocation under deep anesthesia. Blood samples were collected at 4 weeks by heart puncture, and serum samples were stored at −70°C until further analysis.

### 2.3. Dual-Energy X-Ray Absorptiometry (DXA)

The body composition of all the animals was evaluated at the end of the experiment by dual-energy X-ray absorptiometry (Discovery W QDR series, Hologic Inc., Bedford, MA), using the small animal software package. Application of this software had been validated in very young animals as an excellent noninvasive technique for measuring fat and lean tissue in the rat [12]. Total body mass (TBM), lean body mass (LBM), and total body fat were measured. Animals were anesthetized by isoflurane inhalation during examination.

### 2.4. Weighing the Testes

To elucidate the effects of caffeine on gonad growth, both testes were dissected from each rat and cleaned of fat and attached tissues at necropsy. They were then weighed to the nearest 0.001 g with an electronic scale (Adventure Electronic Balances, AR1530, OHAUS Corp., USA) and their morphology was grossly evaluated.

### 2.5. Preparation and Primary Culture of Leydig Cells

To measure testosterone (T_4_) production ex vivo, one testis from each male rat was sliced into four pieces weighing approximately 50~100 mg each. Isolation and purification of Leydig cells followed a similar protocol to that previously described [13]. Briefly, the testis was minced under sterile conditions and the tissue was then dissociated in dissociation buffer containing collagenase D (Roche Applied Science, Indianapolis, IN), DNase I (Roche), and Dispase II (Sigma-Aldrich, St. Louis, MO) at 37°C for 30 min. Following digestion, the seminiferous tubules were allowed to settle and the supernatant was removed. The tubules were then rinsed in DMEM/F12 and allowed to settle and the supernatant was filtered through 100-*μ*m nylon mesh and centrifuged in Percoll gradient buffer. The resulting pellet, containing Leydig cells, was resuspended in DMEM/F12 containing 2% BSA, placed in 24-well plates (3 × 10^5^ cells/well) and cultured with or without luteinizing hormone (200 ng/mL) for 24 h at 37°C, in a 5% CO_2_ incubator. After 24 h of incubation, the medium was collected for T_4_ assay.

### 2.6. Histological Analysis of the Testis

Immediately after removal, one testis from each animal was processed for sectioning. Serial sections of 5 *μ*m thickness were taken from the midportion of each testis and stained with hematoxylin and eosin. All histomorphometric evaluations were performed by the same trained, calibrated, and blinded examiner using an image analysis system (Leica LAS software) coupled to a light microscope (DM4000B, Leica, Heidelberg, Germany) with final magnifications of 100x or 200x. Four serial sections were traced for each testis per animal, and eight measurements per section were made of the number of seminiferous tubules within two defined regions (1.23 mm^2^) at 100-fold magnification and these were combined to obtain a mean value per animal. Eight measurements per section were made for analyzing the diameter of seminiferous tubules or area of intertubular space within the same defined regions (0.307277 mm^2^) at 200-fold magnification, and mean value was calculated. The thickness of the seminiferous epithelium was obtained by subtracting the luminal diameter from the tubule diameter [14] in the same seminiferous tubule and combining the measurements to obtain a mean value per animal.

### 2.7. Testosterone Measurement

T_4_ levels were analyzed in serum samples and in the conditioned media collected from the cultured Leydig cells using a commercially available enzyme-linked immunosorbent assay (ELISA) kit (Cusabio Biotech Co., Ltd., China). Intra- and interassay coefficients of variance were less than 15%, and the limit of detection was 0.06 ng/mL under our conditions. The absorbance was read at 450 nm within 15 min, against a blanking well in ELISA Reader (BioRad, Hercules, CA). All samples were run in duplicate in the same assay and each ELISA was repeated twice.

### 2.8. Statistical Analysis

Data for each group were expressed as mean ± standard deviation. All data were analyzed using SPSS version 10.0 for Windows (SPSS Inc., Chicago, IL). Statistical significance was determined by Kruskal-Wallis one-way analysis of variance for multiple group comparisons. The Mann-Whitney *U* test was used for two-group comparisons.

## 3. Results

### 3.1. Body Weight Gain and Daily Food Intake

**Figure 1:**
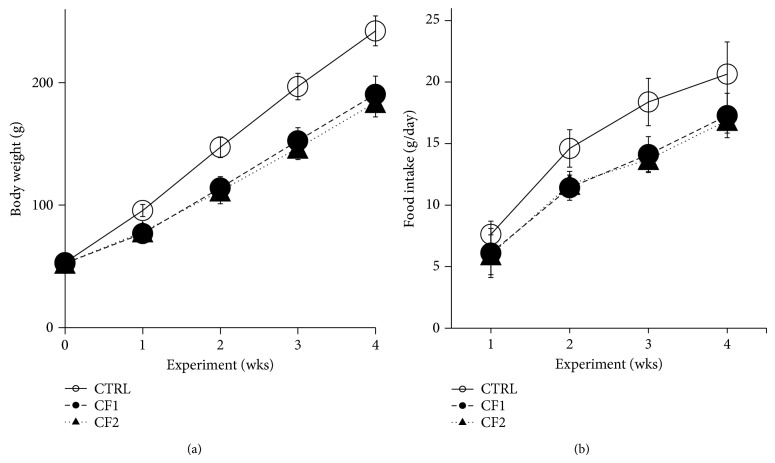
Effect of caffeine exposure on body weight and food intake in immature male rats. (a) Average body weight measured in the rats fed with vehicle or caffeine is depicted every week although body weight was measured daily. (b) Average daily food consumption was calculated by averaging the amount of food consumed over one week and is depicted for each week. Values are expressed as mean ± SD. Open circle, CTRL (control); filled circle, CF1 (caffeine 120 mg/kg/day); filled triangle, CF2 (caffeine 180 mg/kg/day).

As shown in Figure 1(a), all groups gained weight throughout the experimental period. Although there were no differences in starting body weights between the groups (CTRL, 53.1 ± 1.5 g; CF1, 52.6 ± 1.6 g; CF2, 52.6 ± 2.1 g), the caffeine-fed groups weighed significantly less than the controls. The difference was significant from the first week of the experiment and it persisted throughout the experiment. The final body weights of the treatment groups were significantly less than those of the controls (CTRL, 242.2 ± 12.2 g; CF1, 190.5 ± 14.8 g; CF2, 183.8 ± 11.7 g, *p* < 0.001 versus CTRL). However there was no difference between the caffeine groups. Likewise, food consumption was decreased in the caffeine-fed groups from the 1st week of caffeine exposure, although a significant reduction was only attained in the CF2 (*p* < 0.05 versus CTRL). The significant reduction persisted until the end of experiment (*p* < 0.001 versus CTRL) (Figure 1(b)). However, no caffeine dose effect was observed.

### 3.2. Body Composition

**Figure 2:**
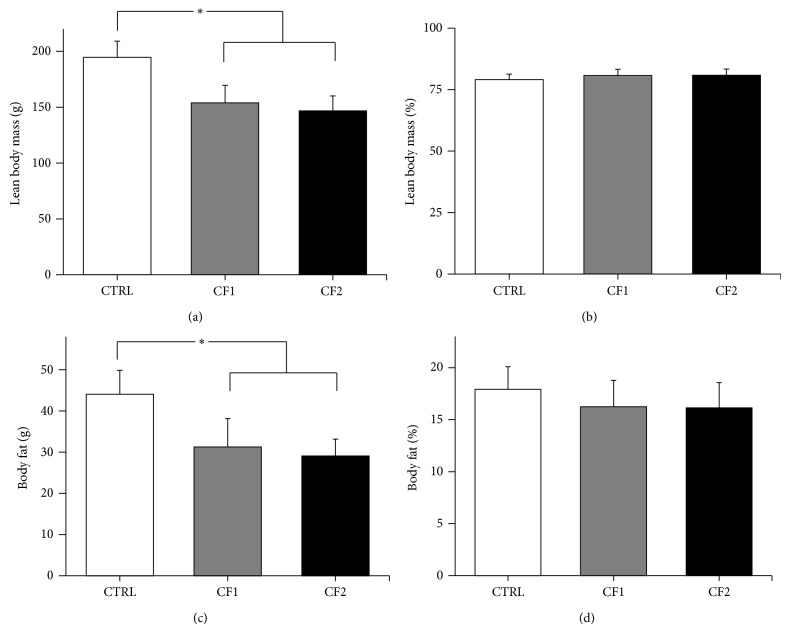
Effect of caffeine exposure on lean body mass and body fat as determined by DXA. Comparison of (a) lean body mass, (b) the percentage of lean body mass, (c) total body fat, and (d) the percentage of body fat between the control and caffeine-fed groups at the end of experiment. Lean body mass (%), lean body mass divided by total body mass; body fat (%), total body fat divided by total body mass. Values are expressed as mean ± SD. CTRL, control; CF1, caffeine 120 mg/kg/day; CF2, caffeine 180 mg/kg/day. ^∗^ *p* < 0.001 versus CTRL.

The data are summarized in Figure 2. Parallel with TBM, body fat and LBM also declined in the caffeine-fed groups compared to the control (*p* < 0.05). However, the percentage of body fat was not significantly different (CTRL versus CF1 versus CF2; 17.9 ± 2.2%, 16.2 ± 2.6%, and 16.1 ± 2.5%), although some decrease was observed in the caffeine-fed groups. Likewise, the percentage of LBM did not differ between the control and caffeine-fed groups, pointing to a proportional reduction in body fat and LBM in the caffeine-fed groups.

### 3.3. Weights of the Testes

**Figure 3:**
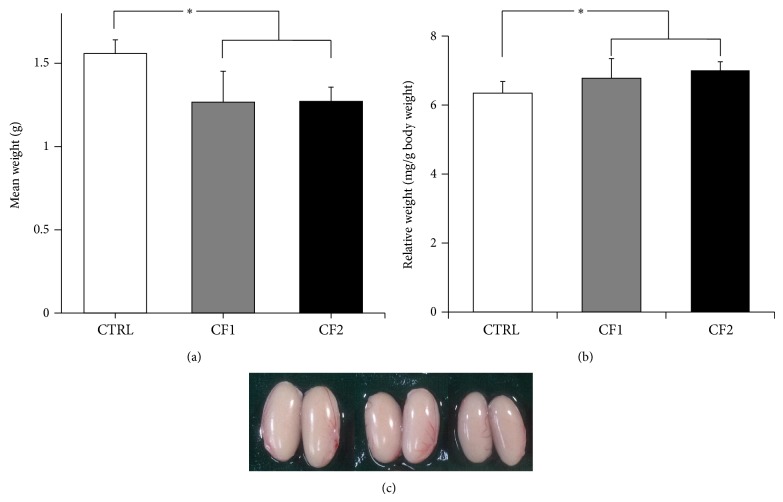
Effect of caffeine on the weights of the testes in the control and caffeine-fed animals. (a) Absolute testes weight (g) and (b) relative testis weight to body weight (mg/g body weight) in the control and caffeine-fed groups at 4 weeks of caffeine exposure. Values are expressed as mean ± SD. ^∗^ *p* < 0.001 versus CTRL. (c) Representative picture of the testes. From the left in order, testes representing each group are designated as CTRL, CF1, and CF2. CTRL, control; CF1, caffeine 120 mg/kg/day; CF2, caffeine 180 mg/kg/day.

To investigate the effect of caffeine on the reproductive organ in vivo, the testes were weighed (Figure 3). As shown in Figure 3(a), absolute testis weight was significantly reduced in the caffeine-fed groups compared to the control (CTRL, 1.56 ± 0.09 g; CF1, 1.27 ± 0.23 g; CF2, 1.27 ± 0.09 g) (*p* < 0.001 versus CTRL). On the contrary, testis weights relative to body weight were significantly increased in caffeine-fed groups (CTRL, 6.35 ± 0.34; CF1, 6.78 ± 0.57; CF2, 7.01 ± 0.25 mg/g body weight) (*p* < 0.001 versus CTRL) (Figure 3(b)). Therefore, reductions in absolute testis weight were not proportional to body weight. Similar data were obtained from an analysis on each testis. However, there was no significant difference between the caffeine-fed groups. As clearly shown in the representative picture (Figure 3(c)), caffeine feeding caused a significant reduction in testes size.

### 3.4. Histological Findings in the Testis

**Figure 4:**
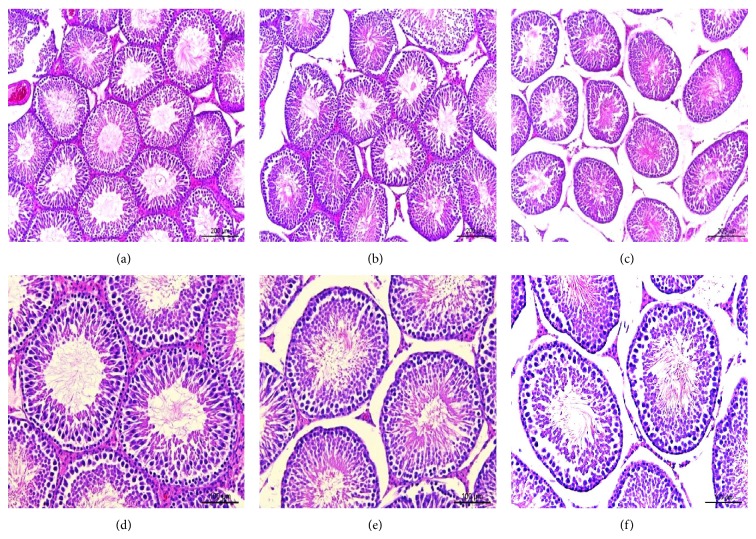
Representative sections of the testis from the control and caffeine-fed groups. The tissues were stained with hematoxylin and eosin. Sections from the control ((a), (d)), CF1 ((b), (e)), and CF2 ((c), (f)). CTRL, control; CF1, caffeine 120 mg/kg/day; CF2, caffeine 180 mg/kg/day. Scale bars: (a)~(c) = 200 *μ*m; (d)~(f) = 100 *μ*m.

The histomorphometric parameters analyzed on histological sections of the testes are summarized in Table 1. Representative testicular sections are shown in Figure 4. Caffeine consumption caused a dose-dependent reduction in the mean diameter of the seminiferous tubules (CTRL, 295.60 ± 14.03; CF1, 280.12 ± 9.05; CF2, 271.82 ± 11.58) (*μ*m) (*p* < 0.01 versus CTRL). Although there was no statistically significant difference between the caffeine-fed groups, mean diameters in CF2 were smaller than in CF1. Also, the height of the germinal epithelium was significantly reduced in CF2 compared to both the control (*p* < 0.001) and CF1 (*p* < 0.01). On the other hand, the numbers of seminiferous tubules counted within the same defined areas were similar in the control and caffeine-fed groups. In contrast, the intertubular area increased dose-dependently in the caffeine-fed groups (*p* < 0.01, CTRL versus CF1; *p* < 0.001, CTRL versus CF2). The histomorphometric changes noted in the caffeine-fed animals most likely contributed to the reduction in testes size and weight.

### 3.5. Testosterone Concentration

**Figure 5:**
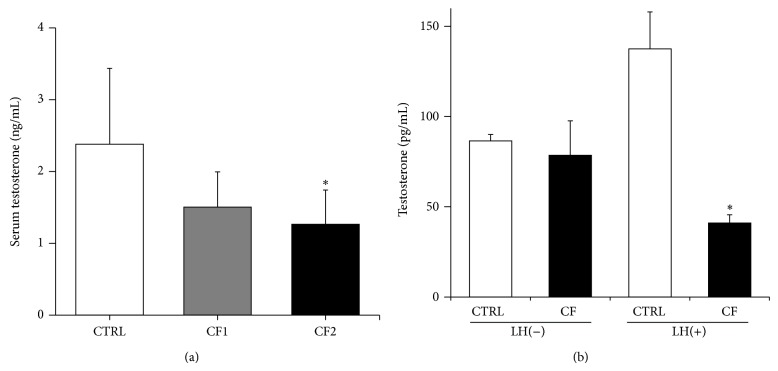
Effect of caffeine on serum testosterone levels and endogenous Leydig cell testosterone production. (a) Serum concentration of testosterone in the control and caffeine-fed groups after 4 weeks of caffeine exposure. Data are presented as mean ± SD based on two independent measurements. CTRL, control; CF1, 120 mg caffeine-fed; CF2, 180 mg caffeine-fed. ^∗^ *p* < 0.05 versus CTRL. (b) Testosterone production with or without LH (200 ng/mL) in rat primary Leydig cells retrieved from the control and caffeine-fed animals at 4 weeks of caffeine exposure. Data are presented as mean ± SD based on two independent measurements. CTRL, Leydig cells retrieved from the control animals; CF, Leydig cells retrieved from caffeine-fed animals (120 mg/kg/day). ^∗^ *p* < 0.05 versus CTRL.

There were lower levels of T_4_ in both caffeine-fed groups, although a significant reduction was attained only at the 180-mg dose (*p* < 0.05 versus CTRL) (Figure 5(a)). Serum levels of T_4_ could be highly variable depending on releasing pulses from the testes into the blood [15]; thus we also analyzed ex vivo T_4_ production from primary Leydig cells retrieved from the caffeine-fed animals (Figure 5(b)). Basal-T_4_ productions were not different between the control and caffeine-fed groups, but stimulated-T_4_ productions were profoundly decreased in Leydig cells retrieved from the caffeine-fed animals compared to those from the control. On the other hand, serum LH levels were also measured at the end of experiment (CTRL: 4.64 ± 2.68, CF1: 6.33 ± 4.28, and CF2: 11.72 ± 14.94 mIU/mL; mean ± SD) and the differences were not significant due to variability.

## 4. Discussion

Our data demonstrate that chronic high doses of caffeine exposure during the pubertal growth period interfere with testis growth and testosterone secretion, accompanied by histomorphometric changes in the testis. Considering that male offspring are more susceptible than females to caffeine treatment during both gestation and lactation [3, 16], we chose male rats to evaluate whether pubertal caffeine exposure has effects similar to prenatal exposure. The choice of dose levels was based on the literature coupled with range finding studies to avoid sublethal effects at the highest dose. The median lethal dose of caffeine administered orally to rats is 192 mg/kg [17]. The doses (120- or 180-mg) chosen here, which have been reported to interfere with components of the endocrine system such as the pituitary and adrenal glands in the fetuses of treated pregnant rats [18], were within the range found to have no undue systemic toxicity in our preliminary study. Currently, body surface area- (BSA-) based dose calculations are the most appropriate method for extrapolating from animals to humans and they are far superior to simple conversions based on body weight [19]. Using dose conversion based on the BSA in humans and rats, the dosages employed in this study were equivalent to approximately 19.4- and 29.1-mg/kg in humans. Most energy drinks provide more than 1 mg/mL of caffeine. Therefore, one or two energy drinks can easily lead to the above doses of caffeine.

A number of human and animal studies have demonstrated that gestational and lactational caffeine exposure causes fetal growth retardation and low birth weight [20]. Likewise, caffeine-fed immature male rats showed a significant reduction in body weight [7]. Consistent with this, we noted a significant reduction in body weight gain in the caffeine-fed animals after only one week (*p* < 0.001 versus CTRL) and these reductions persisted throughout experimental period (Figure 1(a)). In contrast, another study found no difference between the control and caffeine-fed groups [21]. This discrepancy may result from differences in doses, duration, administration route, and age between the studies. Because food intake was persistently decreased after 1 week of caffeine exposure (Figure 1(b)) and caffeine is known to increase metabolic rate and fat oxidation [22], these effects could contribute to the lower body weight gain in the caffeine-fed animals. Also, since body weight and food intake influence the onset of puberty [23], reduced body weight resulting from caffeine exposure could contribute to the delayed pubertal growth.

Increased muscle mass and body fat are two of the major physical changes characterizing normal pubertal development. Previous studies suggest that, more than weight, an appropriate body composition or a certain percentage of fat is needed to start puberty in rats [24]. Along with the poor weight gain in the caffeine-fed groups, LBM and body fat declined significantly (Figure 2). Despite some disagreement, many human studies have pointed to a major influence of body fat on the onset of puberty [25, 26]. For instance, obesity is associated with either early or delayed puberty in boys [25]. Although the reductions of muscle and body fat in the caffeine-fed groups were proportional to the TBM, body fat percentage declined more in the caffeine-fed groups than in the control (CTRL = 17.9 ± 2.2%, CF1 = 16.2 ± 2.6%, and CF2 = 16.1 ± 2.5%). This result suggested that caffeine may inhibit the fat deposition and muscle growth normally occurring during puberty.

Among the major physical alterations of normal pubertal development, changes in testicle size are closely associated with the initiation of puberty in boys [27]. Although appropriate weight and adiposity might be necessary for puberty to occur in girls [26], previous reports showed that a critical body weight does not seem to be essential in males [28, 29]. In addition, rapid testicular growth is stimulated by adequate bioavailability of gonadotropin and testosterone [30–32]. Thus, a negative influence of caffeine on testis growth during the pubertal period may be related to disrupted hormonal milieu. Consistent with our result, maternal caffeine exposure causes a significant dose-dependent reduction in the testis weight of offspring [3]. In contrast, no effect of caffeine exposure on testis weight was noted in adult rats [33]. Considering that puberty is a critical period of sexual maturation, caffeine exposure may cause more adverse effects in this period than in adulthood when development has finished. Thus, peripubertal exposure to caffeine may impact gonadal maturation and later gonadal function.

The testis is largely composed of tightly coiled seminiferous tubules, which contain differentiated germ cells [34, 35]. The size or number of seminiferous tubules could be a main factor to determine the weight of the testis. As expected from the reduction in testis size, the diameter of seminiferous tubules decreased in the caffeine-fed groups (Table 1). Similarly, prenatal caffeine exposure significantly reduced seminiferous tubule diameter [3] and inhibited differentiation of the seminiferous cords [5]. On the other hand, we did not find any difference in the number of seminiferous tubules between groups and this could be explained by the fact that the pubertal period is a period of organic maturation rather than morphogenetic reorganization [5]. Several animal studies have also demonstrated a negative influence of caffeine on the seminiferous tubules, although the dose, duration, administration route, and animal strains were different [36, 37]. As the seminiferous tubule consists of the germinal epithelium including differentiated spermatogenic cells [34, 35], a reduction in seminiferous tubule size points to detrimental changes in the germinal epithelium. In fact, adult animal studies revealed marked damage to the germinal epithelium in the form of degeneration or disruption [38]. Our results also showed a reduction in germinal epithelium height, which may reflect a decreased number of germ cells or differentiated spermatogenic cells. It is known that optimal sperm production does not occur until about 75 days, in the rat [39]. Here, we used immature rats (PD22–50) and sperm analysis was not appropriate for this age. Although we did not perform sperm analysis, a reduction in spermatogenesis can be assumed. In addition, it was demonstrated that prenatal caffeine exposure inhibits differentiation of interstitial tissue and Leydig cells, leading to a significant reduction in Leydig cell number [5]. Like the prenatal effect, we observed histological alteration to the interstitial tissue with loose interstitial tissue and decreased numbers of cells, resulting in significantly increased intertubular distance in the caffeine-fed groups (Figure 4). Consistent with our result, caffeine exposure in adult rat led to degeneration of Leydig cells [34]. In contrast, another study reported Leydig cell hyperplasia in caffeine-fed animals [8]. This discrepancy might be related to caffeine dose because the latter study used an unusually high dose (250 mg/kg/day), which exceeds the lethal dose (192 mg/kg/day) in the rat [17].

Serum concentrations of T_4_ are positively associated with pubertal progression [40]. In addition, adequate circulating T_4_ plays an important role in the structural and functional integrity of the reproductive organs [41]. Therefore, the reduction of the testis weight in the caffeine-fed groups is associated with inadequate circulating male hormone (Figure 5). Prenatal caffeine exposure caused significantly lower T_4_ levels in male human offspring [6] and rats [3]. In agreement with previous work, we found that pubertal caffeine exposure affected T_4_ levels like prenatal exposure. Another study using immature male rats measured T_4_ levels after 4 weeks of caffeine exposure (5, 20, and 100 mg/kg/day) and reported no clear effect [7]. Furthermore, several clinical and animal studies have reported that chronic caffeine consumption increased serum T_4_ levels in adult human [6] and adult animals [42]. The difference may be due to differences in the method of assay, administration protocols, or age of experimental subjects (young or older rats). However, it is clear that caffeine consumption interferes with serum T_4_ levels in immature male rats, contributing to the impaired gonadal maturation and function. On the other hand, decreased T_4_ may also contribute to the reduction in muscle mass in caffeine-fed animals (Figure 2) because it is an important stimulator of skeletal muscle development in men [43]. The decreased serum T_4_ level in caffeine-fed animals may be the result of impaired synthesis or enhanced metabolism. As serum T_4_ is largely produced by the testicular Leydig cells [44], caffeine would be expected to affect androgen-producing cells. An in vitro study reported that caffeine increased T_4_ secretion in a cell line and primary rat Leydig cells [7]. Because in vitro study would not reflect precisely in vivo status, we used cells retrieved from treated animals to measure secretory activity more precisely. As shown in Figure 5, a blunted response to LH stimulation was noted in Leydig cells from the caffeine-fed groups. It has been reported that prenatal caffeine exposure significantly inhibited the enzyme activity of Leydig cells relevant to androgen production [5]. In addition, an animal study suggested a direct effect of caffeine on the testis instead of an indirect effect via alterations of LH secretion [5]. Therefore, pubertal caffeine exposure may directly affect Leydig cells possibly by alteration of enzyme activity and result in a reduction in circulating T_4_ levels.

In conclusion, caffeine may interfere with testicular steroidogenesis, particularly during puberty and sexual maturation, although the mechanism underlying the effects of caffeine on the Leydig cells in immature animals remains to be determined. Until now, there have been no data on the interaction between the Leydig cells, T_4_, and caffeine exposure during the puberty. Our results demonstrate that caffeine can interfere with the endogenous T_4_ secretion from the Leydig cells, possibly by reducing the sensitivity of the Leydig cells to gonadotrophic stimulation. In addition, we confirmed that pubertal administration of caffeine reduced testis growth and altered testis histomorphology.

## Figures and Tables

**Table 1:** Histomorphometric parameters analyzed in the testes of the control and caffeine-fed groups.

Parameter	CTRL (*N* = 15)	CF1 (*N* = 15)	CF2 (*N* = 15)
Seminiferous tubule diameter (*μ*m)	295.6 ± 14.03	280.1 ± 9.05^∗^	271.8 ± 11.58^∗∗^
Germinal epithelium height (*μ*m)	167.3 ± 6.77	163.8 ± 7.15	153.6 ± 8.2^∗∗,†^
Seminiferous tubule number	60 ± 4.6	61 ± 2.8	63 ± 6.5
Intertubular area (mm^2^)	0.07 ± 0.02	0.10 ± 0.03^∗^	0.11 ± 0.01^∗∗^

Values are expressed as mean ± SD. ^∗^ *p* < 0.01, ^∗∗^ *p* < 0.001 versus CTRL; ^†^ *p* < 0.01 CF1 versus CF2. CTRL: control; CF1: caffeine 120 mg/kg/day; CF2: caffeine 180 mg/kg/day.
